# Frequency of Citrus Fruit Intake Is Associated With the Incidence of Cardiovascular Disease: The Jichi Medical School Cohort Study

**DOI:** 10.2188/jea.JE20100084

**Published:** 2011-05-05

**Authors:** Tomoyo Yamada, Shinya Hayasaka, Yosuke Shibata, Toshiyuki Ojima, Tomohiro Saegusa, Tadao Gotoh, Shizukiyo Ishikawa, Yosikazu Nakamura, Kazunori Kayaba

**Affiliations:** 1 Department of Community Health and Preventive Medicine, Hamamatsu University School of Medicine, Hamamatsu, Japan; 2 Hamamatsu Municipal National Health Insurance Sakuma Hospital, Hamamatsu, Japan; 3 Wara National Health Insurance Clinic, Gujyo, Gifu, Japan; 4 Division of Community and Family Medicine, Center of Community Medicine, Jichi Medical University, Shimotsuke, Tochigi, Japan; 5 Department of Public Health, Jichi Medical University, Shimotsuke, Tochigi, Japan; 6 School of Health and Social Services, Saitama Prefectural University, Koshigaya, Saitama, Japan

**Keywords:** citrus fruit, cardiovascular disease, cerebral infarction, cohort studies, Japan

## Abstract

**Background:**

It has been reported that fruit intake protects against cardiovascular disease (CVD). However, most of the relevant studies were conducted in Western countries, and only a few investigated Japanese populations. The present cohort study assessed the effect of citrus fruit intake on the incidence of CVD and its subtypes in a Japanese population.

**Methods:**

A baseline examination consisting of physical and blood examinations and a self-administered questionnaire was conducted during the period from April 1992 through July 1995. Dietary habits were assessed using a food frequency questionnaire that was divided into 5 categories. Citrus fruit was examined separately due to its frequent consumption by the general Japanese population. Using the Cox proportional hazards model, data from 10 623 participants (4147 men, 6476 women) who had no history of CVD or carcinoma were analyzed to assess the association between frequency of citrus fruit intake and CVD incidence.

**Results:**

Frequent intake of citrus fruit was associated with a lower incidence of CVD: the hazard ratio for almost daily intake versus infrequent intake of citrus fruit was 0.57 (95% confidence interval: 0.33–1.01, *P* for trend = 0.04) in men and 0.51 (0.29–0.88, *P* for trend = 0.02) in women. Frequent intake of citrus fruit was also associated with lower incidences of both all stroke and cerebral infarction, but not hemorrhagic stroke or myocardial infarction.

**Conclusions:**

Frequent intake of citrus fruit may reduce the incidence of CVD, especially cerebral infarction, in men and women.

## INTRODUCTION

Cardiovascular disease (CVD) is a major cause of death in developed countries. In the period since World War II, the distribution of diseases in Japan has dramatically changed and has begun to resemble that of Western countries. More recently, an important research theme has been to identify methods that might reduce CVD by using preventive means such as diet.

Fruit intake has been reported to protect against CVD. Fruit contains vitamins (such as vitamin C and folate), carotenoids, flavonoids, potassium, and fiber. Reports have shown that these protect against hypertension^1^^,^^2^ and atherosclerosis by inhibiting low-density lipoprotein oxidation,^3^ preventing increases in homocysteine and platelet aggregation,^3^^,^^4^ and improving glucose intolerance.^2^ High fruit intake has also been associated with reduced CVD mortality^5^^–^^12^ and incidence.^13^^–^^17^ Most of these studies were conducted in Western countries, however. In Japanese cohorts, 3 prospective studies^9^^–^^11^ reported a reduction in CVD mortality associated with frequent fruit intake, and 2 prospective studies^14^^,^^17^ noted a reduction in CVD incidence associated with fruit intake in Japanese populations. CVD encompasses a number of conditions that affect the cardiovascular system, such as coronary heart disease, stroke, and heart failure, and the etiologies and resulting pathologic conditions of these CVD subtypes vary. However, little research has attempted to assess the effect of fruit intake on these subtypes.^11^^,^^14^^,^^18^ The present study was carried out to examine the potential protective effects of fruit intake on the incidence of CVD and its subtypes in a Japanese population.

In Japan, citrus fruit accounted for 32.6% of annual all-fruit consumption in 2008, and it has been the most widely distributed type of fruit in Japan for more than 20 years, according to the Ministry of Agriculture, Forestry and Fisheries.^19^ Because it is the most popular fruit type in Japan, we specifically evaluated citrus fruit intake and assessed its protective effects against CVD incidence.

## METHODS

### Study population

We evaluated data from the Jichi Medical School (JMS) Cohort Study, which previously investigated the risk factors for CVD. This study enrolled 12 490 participants (4911 men, 7579 women) from 12 communities across Japan. In Japan, a mass screening examination for CVD has been conducted since 1982 in accordance with a system established by the Health and Medical Service Law for the Aged. This system was used to collect the baseline data for the present study. A municipal government office in each community site sent a personal invitation letter by mail or provided public information. Baseline examinations were conducted from April 1992 through July 1995 and consisted of physical and blood examinations and a self-administered questionnaire. The questionnaire was designed to obtain information on anthropometric and lifestyle exposures and dietary intake. A detailed description of the standardized collection of baseline examinations was published previously by Ishikawa et al.^20^ Of the 12 490 participants, 95 declined follow-up and 7 could not be contacted after baseline examination. Thus, a total of 4869 men and 7519 women were followed; the follow-up rate was 99.2%. We excluded participants with a history of CVD or carcinoma and those with missing data on fruit intake. Ultimately, data from 10 623 participants (4147 men, 6476 women) were analyzed in the present study.

### Baseline examination

#### Physical and blood examinations

In all communities, physical examinations took place using similar protocols. All participants measured their body height without shoes. Body weight while fully clothed was recorded by subtracting 0.5 kg (in the summer) or 1 kg (in other seasons) from the recorded weight to account for clothing. Body mass index (BMI) was defined as body weight in kilograms divided by the square of body height in meters. Systolic blood pressure (SBP) was measured using the same type of fully automated sphygmomanometer, which was placed on the right arm of the participant after they had been sitting for 5 minutes. Serum cholesterol concentration was measured by taking a blood sample from the antecubital vein of seated participants. Total cholesterol (TC) and triglycerides (TG) were measured using an enzymatic method, and high-density lipoprotein (HDL) cholesterol was measured using the phosphotungstate precipitation method (Wako, Osaka, Japan; interassay coefficient of variation, 1.5%).

#### Anthropometric and lifestyle exposures

Further lifestyle- and health-related variables were collected using self-reported questionnaires. Physical activity index (PAI) was calculated on the basis of the criteria included in the Framingham Study.^21^ The total hours of sleeping, working, and leisure time within a day were multiplied by weight, based on the oxygen consumption required for each activity. Smoking status was classified as never smoker, ex-smoker, or current smoker, and alcohol consumption was categorized as never drinker, ex-drinker, or current drinker. Education level was assessed by age at completion of the highest educational qualification. Marital status was classified as currently married or unmarried.

#### Dietary habits

Dietary habits were assessed using a food frequency questionnaire (FFQ) that contained 30 items, including 2 subgroups on fruit consumption, namely, citrus fruit, such as satsuma mandarins (*mikans*), and other fruits. At the beginning of the FFQ, we asked participants to describe their usual diet. They indicated how often they consumed foods daily by answering from 5 multiple choice options: 1 = infrequent, 2 = 1–2 times/month, 3 = 1–2 times/week, 4 = 3–4 times/week, and 5 = almost daily. Although there were other questions that assessed juice consumption (using the 5 categories in the FFQ), there was no mention in the FFQ of the type of juice consumed. Because of this, we excluded fruit juice consumption from our estimation of citrus fruit intake. The FFQ was based on the one used in the Japan Collaborative Cohort (JACC) Study, and the reproducibility and validity of the intake frequency were examined previously.^22^ To test the reproducibility of intake frequency, the 2 FFQs were distributed at 1-year intervals. The validity of the intake frequency of the FFQ was assessed by conducting a weighted dietary record.

### Follow-up

The mass screening examination system that had been conducted each year was used to follow participants. They were asked whether they had a history of CVD at the mass screening examination each year. Those with such a history were asked which hospital they had visited and when. Participants who did not attend the screening examination were contacted by mail or telephone. Public health nurses visited the homes of the participants to obtain additional information when necessary. Death certificates were collected from public health centers with the official permission of the Agency of General Affairs and the Ministry of Health, Labour and Welfare. We stopped follow-up of participants who died before the end of the study. Death from CVD was counted in the CVD incidence data. Information concerning participants who moved out of the study area during the follow-up period was obtained annually from the relevant municipal government; these participants (*n* = 340) were no longer followed from the day they left the study area. The mean duration of follow-up, ie, from the time of the baseline examination until either CVD incidence, relocation to another area, or the end of the study, was 10.7 years.

### Diagnostic criteria

CVD was defined as stroke, myocardial infarction, or sudden death, whichever occurred first. If a CVD event was suspected, we requested duplicate computer tomography scans or magnetic resonance images (in cases of stroke) and electrocardiograms (in cases of myocardial infarction). The diagnoses were determined independently by a diagnosis committee composed of a radiologist, a neurologist, and 2 cardiologists. A diagnosis of stroke was defined as sudden onset of a focal, nonconvulsive neurological deficit persisting longer than 24 hours. Stroke subtype was classified as cerebral infarction, hemorrhagic stroke, or undetermined, according to the criteria of the National Institute of Neurological Disorders and Stroke.^23^ Myocardial infarction was diagnosed according to the criteria of the World Health Organization Multinational Monitoring of Trends and Determinants in Cardiovascular Disease (MONICA) Project.^24^

### Statistical analysis

All analyses were performed separately for men and women using the Statistical Package for Social Science (SPSS) for Windows (SPSS Japan Inc., version 15.0, Tokyo, Japan). First, general characteristics were categorized by frequency of citrus fruit intake by using means (standard deviation) and proportions. Next, to clarify the associations between frequency of citrus fruit intake and potential confounders, *P* values were calculated using 1-way analysis of variance and the chi-square test for variables. Finally, a Cox proportional hazards model was used to calculate the hazard ratio (HR) and 95% confidence interval (CI) of the incidence of CVD in relation to the frequency of citrus fruit intake, with adjustment for age and study area (HR1), or adjustment for age, study area, BMI, SBP, TC, PAI, smoking status, alcohol consumption, education level, and marital status (HR2). Age, BMI, SBP, TC, and PAI were entered in the model as continuous variables; study area (12 areas), smoking states (current, ex-, or never smoker), alcohol consumption (current, ex-, or never drinker), education level (younger than 16 years or not at age of completion), and marital status (married or not) were entered as categorical variables.

## RESULTS

During an average follow-up period of 10.7 years, we documented 488 CVD events (270 in men, 218 in women): 383 strokes (201 in men, 182 in women)—including 249 cerebral infarctions (146 in men, 103 in women) and 133 hemorrhagic strokes (55 in men, 78 in women)—and 76 myocardial infarctions (53 in men, 23 in women). These events included 301 deaths from CVD (75 in men, 64 in women): 74 from stroke (38 in men, 36 in women)—including 32 deaths from cerebral infarction (18 in men, 14 in women) and 41 deaths from hemorrhagic stroke (20 in men, 21 in women)—and 15 from myocardial infarction (10 in men, 5 in women).

The baseline characteristics of participants according to frequency of citrus fruit intake are shown in Table 1. In both men and women, frequent consumers of citrus fruit tended to be older and less likely to smoke and drink. Among men, higher citrus fruit intake was associated with less physical activity and being married. Women with a high citrus fruit intake were better educated and had a higher BMI, SBP, and serum concentrations of TC and TG. However, the distributions of BMI, SBP, and serum TC and TG concentrations were within approximately normal ranges.

**Table 1.** Baseline relationships between frequency of citrus fruit intake and potential confounders

	Frequency of citrus fruit intake^a^					*P*	Frequency of citrus fruit intake^a^					*P*
		
	1 (low)	2	3	4	5 (high)		1 (low)	2	3	4	5 (high)	
	Men						Women					
No. of subjects	570	1096	1445	704	332		466	987	2092	1749	1182	
Age, y (mean ± SD)	53.2 (13.1)	52.4 (12.6)	55.0 (12.1)	57.7 (10.1)	58.7 (9.7)	<0.01	54.2 (12.9)	53.0 (12.8)	54.1 (11.9)	55.5 (10.3)	59.2 (9.0)	<0.01
Body mass index, kg/m^2^ (mean ± SD)	22.8 (3.0)	23.0 (2.8)	22.9 (2.9)	23.0 (2.8)	23.0 (2.7)	0.81	22.9 (3.4)	23.0 (3.3)	23.0 (3.2)	23.0 (3.1)	23.5 (3.1)	0.001
Systolic blood pressure, mm Hg (mean ± SD)	131 (21.0)	131 (20.8)	131 (20.1)	132 (19.6)	130 (21.1)	0.27	128 (22.7)	127 (21.5)	127 (21.3)	128 (20.3)	130 (20.7)	0.02
Serum cholesterol concentration												
Total cholesterol, mg/dl (mean ± SD)	183 (35.0)	183 (34.9)	186 (34.9)	187 (32.3)	184 (34.1)	0.02	193 (36.3)	193 (35.1)	195 (33.6)	198 (35.1)	202 (34.7)	<0.01
High-density lipoprotein, mg/dl ​ (mean ± SD)	49 (13.6)	49 (14.2)	49 (13.2)	49 (12.9)	48 (13.0)	0.50	53 (12.7)	53 (12.9)	53 (12.7)	53 (12.2)	52 (12.1)	0.69
Triglyceride, mg/dl (mean ± SD)	130 (90.2)	127 (89.5)	126 (78.3)	128 (91.0)	129 (91.5)	0.86	101 (55.4)	105 (63.6)	105 (62.3)	109 (65.5)	119 (73.8)	<0.01
Physical activity index (mean ± SD)	36.2 (10.3)	35.8 (9.5)	35.8 (9.6)	35.7 (9.0)	34.1 (8.3)	0.02	31.8 (6.3)	31.2 (5.4)	31.7 (5.6)	31.7 (5.3)	31.5 (5.1)	0.09
Current smoker, %	64.4	55.3	48.7	44.7	41.3	<0.01	10.1	10.0	5.3	3.8	3.7	<0.01
Current alcohol drinker, %	74.9	77.3	77.5	72.4	69.9	0.01	28.2	33.5	27.1	22.0	19.9	<0.01
Education level (age at completion)												
≤15 years, %	49.3	43.6	41.7	44.8	43.1	0.04	59.3	51.0	48.6	49.1	51.9	0.001
Married, %	86.9	89.7	92.2	95.1	94.5	<0.01	89.4	91.5	91.9	92.3	90.8	0.26

^a^1 = infrequent, 2 = 1–2 times/month, 3 = 1–2 times/week, 4 = 3–4 times/week, and 5 = almost daily.

As shown in Tables 2 and 3, the risks of CVD were lower in participants who frequently consumed citrus fruit, and there was a clear inverse relationship (HR for almost daily versus infrequent citrus fruit intake: 0.57, 95% CI: 0.33–1.01, *P* for trend = 0.04 in men; and 0.51, 0.29–0.88, *P* for trend = 0.02 in women). A stronger inverse relationship was found between frequency of citrus fruit intake and all-stroke risk in both men (HR for almost daily versus infrequent citrus fruit intake: 0.40, 0.20–0.81, *P* for trend = 0.01) and women (0.47, 0.26–0.87, *P* for trend = 0.02). Regarding stroke subtype, frequent intake of citrus fruit was significantly inversely associated with the risk of cerebral infarction, but not hemorrhagic stroke. There was no significant relationship between frequency of citrus fruit intake and incidence of myocardial infarction. The associations remained after multivariate adjustment.

**Table 2.** Hazard ratios and 95% confidence intervals according to frequency of citrus fruit intake adjusted for potential confounders (men)

	Frequency of citrus fruit intake					*P* for trend
	
	Infrequent	1–2 times/month	1–2 times/week	3–4 times/week	Almost daily	
Person-years	5785	11 847	15 333	7677	3511	
Cardiovascular disease						
No. of cases	47	60	98	44	21	
HR1^a^ (95% CI^b^)	1.00	0.67 (0.46–0.99)	0.73 (0.51–1.04)	0.57 (0.38–0.87)	0.58 (0.35–0.97)	0.02
HR2^c^ (95% CI)	1.00	0.70 (0.47–1.05)	0.75 (0.52–1.09)	0.63 (0.41–0.97)	0.57 (0.33–1.01)	0.04
All-stroke						
No. of cases	38	44	73	33	13	
HR1 (95% CI)	1.00	0.60 (0.39–0.93)	0.66 (0.45–0.98)	0.52 (0.33–0.83)	0.43 (0.23–0.80)	0.01
HR2 (95% CI)	1.00	0.61 (0.39–0.96)	0.68 (0.45–1.03)	0.57 (0.35–0.92)	0.40 (0.20–0.81)	0.01
Cerebral infarction						
No. of cases	27	32	56	24	7	
HR1 (95% CI)	1.00	0.61 (0.37–1.02)	0.71 (0.45–1.12)	0.53 (0.31–0.92)	0.32 (0.14–0.75)	0.008
HR2 (95% CI)	1.00	0.65 (0.38–1.11)	0.73 (0.45–1.18)	0.62 (0.35–1.08)	0.28 (0.11–0.72)	0.02
Hemorrhagic stroke						
No. of cases	11	12	17	9	6	
HR1 (95% CI)	1.00	0.57 (0.25–1.29)	0.54 (0.25–1.15)	0.49 (0.20–1.19)	0.68 (0.25–1.83)	0.30
HR2 (95% CI)	1.00	0.52 (0.22–1.25)	0.57 (0.26–1.25)	0.45 (0.17–1.20)	0.71 (0.24–2.11)	0.36
Myocardial infarction						
No. of cases	10	10	16	10	7	
HR1 (95% CI)	1.00	0.57 (0.23–1.39)	0.62 (0.27–1.39)	0.68 (0.27–1.67)	1.01 (0.38–2.73)	0.91
HR2 (95% CI)	1.00	0.60 (0.25–1.49)	0.62 (0.27–1.43)	0.75 (0.30–1.86)	0.99 (0.34–2.80)	0.95

^a^Hazard ratio adjusted for age and study area.

^b^Confidence interval.

^c^Hazard ratio adjusted for age, study area, body mass index, systolic blood pressure, total cholesterol concentration, physical activity index, smoking status, alcohol consumption, education level, and marital status.

**Table 3.** Hazard ratios and 95% confidence intervals according to frequency of citrus fruit intake adjusted for potential confounders (women)

	Frequency of citrus fruit intake					*P* for trend
	
	Infrequent	1–2 times/month	1–2 times/week	3–4 times/week	Almost daily	
Person-years	5007	10 617	22 994	19 623	13 019	
Cardiovascular disease						
No. of cases	24	44	63	56	31	
HR1^a^ (95% CI^b^)	1.00	1.00 (0.61–1.64)	0.66 (0.41–1.06)	0.69 (0.43–1.11)	0.48 (0.28–0.83)	0.001
HR2^c^ (95% CI)	1.00	0.83 (0.49–1.41)	0.68 (0.42–1.11)	0.75 (0.46–1.22)	0.51 (0.29–0.88)	0.02
All-stroke						
No. of cases	20	37	53	47	25	
HR1 (95% CI)	1.00	1.00 (0.58–1.72)	0.66 (0.39–1.10)	0.68 (0.40–1.14)	0.46 (0.25–0.83)	0.002
HR2 (95% CI)	1.00	0.84 (0.47–1.49)	0.67 (0.39–1.14)	0.73 (0.42–1.25)	0.47 (0.26–0.87)	0.02
Cerebral infarction						
No. of cases	10	23	29	30	11	
HR1 (95% CI)	1.00	1.29 (0.61–2.71)	0.77 (0.37–1.58)	0.94 (0.46–1.93)	0.43 (0.18–1.02)	0.02
HR2 (95% CI)	1.00	1.04 (0.47–2.33)	0.80 (0.37–1.73)	1.02 (0.48–2.20)	0.39 (0.15–1.00)	0.07
Hemorrhagic stroke						
No. of cases	10	14	23	17	14	
HR1 (95% CI)	1.00	0.72 (0.32–1.63)	0.54 (0.26–1.13)	0.45 (0.21–0.99)	0.49 (0.22–1.10)	0.05
HR2 (95% CI)	1.00	0.66 (0.29–1.52)	0.53 (0.25–1.13)	0.49 (0.22–1.08)	0.55 (0.24–1.23)	0.16
Myocardial infarction						
No. of cases	2	3	10	5	3	
HR1 (95% CI)	1.00	0.82 (0.14–4.93)	1.27 (0.28–5.84)	0.76 (0.15–4.00)	0.59 (0.10–3.54)	0.45
HR2 (95% CI)	1.00	0.83 (0.14–4.98)	1.47 (0.32–6.84)	0.84 (0.16–4.46)	0.67 (0.11–4.15)	0.57

^a^Hazard ratio adjusted for age and study area.

^b^Confidence interval.

^c^Hazard ratio adjusted for age, study area, body mass index, systolic blood pressure, total cholesterol concentration, physical activity index, smoking status, alcohol consumption, education level, and marital status.

## DISCUSSION

The present results indicate that frequent consumption of citrus fruit is significantly inversely associated with CVD incidence. In particular, there were marked reductions in all-stroke and cerebral infarction risk, but not in hemorrhagic stroke risk. Frequency of citrus fruit intake was not associated with incidence of myocardial infarction.

As compared with reports that showed a reduction in the incidence of CVD^15^ or cerebral infarction^13^^,^^14^ based on fruit intake, the reductions found in the present study were statistically significant. Yokoyama et al^14^ reported that the multivariate HR of cerebral infarction incidence was 0.51 (95% CI: 0.24–1.10, *P* = 0.07) among women consuming fruit 6 to 7 days per week, as compared with those consuming it 0 to 2 days per week. In the present study, the multivariate HRs of cerebral infarction incidence for almost daily intake versus infrequent intake were 0.28 (95% CI: 0.11–0.72, *P* for trend = 0.02) in men and 0.39 (0.15–1.00, *P* for trend = 0.07) in women. A possible reason for this difference is the present study’s focus on the effects of citrus fruit. In Japan, in addition to citrus fruit, apples, bananas, watermelons, and pears are commonly consumed. However, as compared with these other fruits, citrus fruit is richer in antioxidants such as vitamin C and β-cryptoxanthin.^25^^,^^26^ These antioxidants may protect against CVD or cerebral infarction and contribute to a marked reduction in incidence levels. As compared with the results from previous studies of the association of citrus fruit intake with the incidences of CVD^16^^,^^17^ and cerebral infarction,^13^ the reductions in these incidences associated with frequent citrus fruit intake were more marked in the present analysis. It is possible that the variation in results between studies was due to differences in the categories used to assess the frequency of citrus fruit intake. Nevertheless, our results are generally consistent with findings from prior studies. In addition, our finding of no significant association between the frequency of citrus fruit intake and hemorrhagic stroke incidence is in line with the report by Yokoyama et al.^14^ Although a number of mechanisms by which fruit protects against CVD have been reported,^1^^–^^4^ the results of the present study (ie, that citrus fruit intake is inversely associated with CVD, especially cerebral infarction, but not hemorrhagic stroke) support the hypothesis that antioxidants contained in citrus fruit have beneficial effects on atherosclerosis by inhibiting oxidant formation, reducing oxidized low-density lipoprotein, and repairing oxidant-induced injury.^27^^,^^28^ In the present population, high fruit intake was not cross-sectionally associated with cholesterol concentration. Thus, whether high citrus fruit intake lowers serum cholesterol concentration is not clear from the present results. We recruited participants for mass screening in such a way as to ensure that serum cholesterol values would be distributed almost normally, as was also the case for BMI and blood pressure.

In the present study, the incidence of myocardial infarction was not reduced by frequent intake of citrus fruit. Studies in Western countries^15^^,^^29^^,^^30^ have reported that there is an inverse relationship between the incidence of coronary heart disease and frequent consumption of fruit. In addition, Pereira et al^31^ reported that consumption of dietary fiber from fruit was inversely associated with risk of coronary heart disease. In contrast, there was no reduction in the risk of coronary heart disease by fruit intake in the present study or in another study of Japanese.^11^ Japanese have a lower incidence of myocardial infarction than individuals living in Western countries. This difference in disease structure, and the lower number cases, may have an effect on the results.

Our results show an inverse trend between CVD incidence and fruit intake among both men and women. There are few epidemiologic studies that show a protective effect of fruit intake against CVD risk in men,^6^^,^^9^^,^^18^ although the effect in women has been more frequently documented.^10^^,^^13^^–^^15^^,^^17^ It has been reported that men require much higher intakes of fruit to maintain serum vitamin C and carotenoid concentrations that are identical to those of women.^1^^,^^32^ In addition, as compared with women, men have a substantially heavier burden of CVD risk factors, such as smoking, obesity, and low HDL concentration.^33^ These observations may explain why there is a stronger inverse interaction between fruit intake and CVD risk among women than men. Our results indicate that fruit intake is protective in women and men, despite the potentially higher risk of CVD in the latter.

Current alcohol drinkers and cigarette smokers are exposed to reactive oxidants and free radicals due to their alcohol and cigarette consumption.^34^^,^^35^ It has been extensively reported that serum vitamin C and carotenoid concentrations are low in alcohol drinkers and cigarette smokers.^1^^,^^14^^,^^32^ Moreover, Sugiura et al^32^ indicated that synergic depletion of serum carotenoid concentrations occurs as a result of alcohol consumption and cigarette smoking, after taking into account dietary carotenoid intake. We performed analyses on multiplicative interactions stratified by alcohol consumption and smoking status (data not shown). The results indicated a slight protective effect among men who were current drinkers and smokers, but the interaction was not statistically significant.

Fruit consumption and distribution vary by season. Satsuma mandarins (*mikans*), which account for more than half of citrus fruits distributed in Japan, are eaten mainly between October and March.^19^ Overall, 87.4% of the present participants answered the FFQ between April and September, which is the period when there is less citrus fruit available. Therefore, this FFQ was less likely to have been affected by seasonal variation in citrus fruit intake. The inverse relationship between frequency of citrus fruit intake and CVD incidence did not differ when we analyzed the limited population that underwent baseline examination during the period from April through September (data not shown).

Our study has several potential limitations. First, we did not examine long-term dietary habits. The FFQ was conducted only once. The possibility that dietary habits changed during follow-up cannot be ruled out. Second, the present analysis is not quantitative, and the FFQ was self-reported; thus, the accuracy of the assessment of citrus fruit intake was limited. However, the reproducibility and validity of the FFQ were previously found to be acceptable.^22^ We therefore consider the results of the present study to be reliable. Determining the amount of citrus fruit intake that is sufficient to protect individuals from CVD risk would be useful when providing advice on healthy dietary habits. Further studies are therefore warranted. Third, the potential effect of confounding variables, except those included in our analyses, cannot be totally excluded. In the present study, the participants who frequently consumed citrus fruit were older, had healthier overall behavior, and were more highly educated. These differences in baseline characteristics suggest the possibility that there were residual confounding variables that were incompletely measured. In addition to habitual variables, there are possibly dietary confounding variables, such as energy, saturated fat, fruit juice intake, and supplement use, about which, unfortunately, we have no information.

In conclusion, the present study indicates that frequent intake of citrus fruit may reduce CVD incidence—in particular, cerebral infarction—in both men and women. Citrus fruit consumption has diminished in recent years in Japan,^19^ and this reduction in citrus fruit intake might partly contribute to the increasing number of CVD cases in Japan. Our results show that there are grounds for recommending that citrus fruit intake be increased in order to protect against CVD incidence.
